# A15 GENETIC OR DIET-ASSOCIATED DEFECTS IN MUCUS FACILITATE ULCERATIVE COLITIS PATHOBIONT-DRIVEN COLITIS

**DOI:** 10.1093/jcag/gwac036.015

**Published:** 2023-03-07

**Authors:** H Yang, X Han, C Ma, H Yu, B Vallance

**Affiliations:** Pediatrics, BC Children's Hospital, Vancouver, Canada

## Abstract

**Background:**

The Inflammatory Bowel Diseases (IBD), Crohn’s Disease (CD) and ulcerative colitis (UC) affect > 270,000 Canadians and are increasing in incidence. Ileal CD has been linked to the overgrowth of mucosal adherent *E. coli*. Recent studies have also implicated the adherence of *Escherichia coli* pathobionts to the colonic mucosa of UC patients. Using the representative UC *E. coli* pathobiont p19A, we recently demonstrated it aggravated chemical-induced colitis in susceptible mice, through the actions of the toxin alpha-hemolysin, and by adhering to the inflamed colonic mucosa via the adhesin FimH. It is less clear what host factors control susceptibility to the UC pathobionts. One of the potential candidates is the glycosylated mucin (Muc2) which forms the mucus layer that covers the colonic epithelium and is often impaired in UC patients.

**Purpose:**

To define the role of mucus structure and function in determining susceptibility to the p19A pathobiont, and its ability to cause colitis in mice.

**Method:**

In vitro growth was assessed to test p19A’s ability to utilize mucin-derived sugars as nutrients. Susceptibility to p19A was tested in two mouse models of colonic mucus impairment. The first is a mouse strain deficient in core 1 derived O-glycans in their intestinal epithelial cells (IEC *C1galt1*^-/-^), resulting in reduced Muc2 glycosylation, and thus a thin and impaired mucus barrier. The second model involves feeding wildtype (WT) C57BL/6CR mice a fiber-free (FF) diet, resulting in a significantly thinner colonic mucus layer. The mice were subsequently orally gavaged with p19A and their susceptibility determined by p19A burdens, intestinal histopathology, inflammatory cytokine and short chain fatty acid (SCFA) production.

**Result(s):**

When tested in vitro, the p19A pathobiont was found to use an array of mucin-derived sugars as sole carbon source to proliferate. Following oral gavage of WT mice fed a normal diet, immunostaining identified p19A within the colonic mucus but it did not reach the colonic mucosa or cause disease. In contrast, p19A was found at the colonic mucosal surface of mucus-defective IEC *C1galt1*^-/-^ mice (as compared to IEC *C1galt1*^flox/flox^ mice) and in WT mice fed a fiber-free diet. This mucosal adherence was associated with increased body weight loss during the course of infection, as well as increased p19A burdens, colonic pathology and pro-inflammatory cytokine expression. Especially fiber-free diet-fed mice showed reduced SCFA levels in their feces at baseline. When the mice were given exogenous SCFA (tributyrin) orally, p19A infection was reduced.

**Conclusion(s):**

Our results indicate that UC *E. coli* pathobionts are able to dwell within colonic mucus and utilize mucin sugars as nutrients. Moreover, they can reach the mucosal surface and induce colitis in hosts suffering genetic or diet-based mucus dysfunction. In part, this susceptibility reflects the important role played by mucus in the production of SCFA, suggesting potential therapeutic approaches for patients suffering UC.

**Please acknowledge all funding agencies by checking the applicable boxes below:**

CCC, CIHR

**Disclosure of Interest:**

None Declared

